# Supplementary material for: Bacterial population dynamics during colonization of solid tumors
Source: Mol Syst Biol. 2025 Dec 15;22(3):412–34. doi: 10.1038/s44320-025-00175-5 (PMC12953616; doi:10.1038/s44320-025-00175-5)
Supplement: Supplementary file 12 — Expanded View Figures [file 44320_2025_175_MOESM12_ESM.pdf]

Expanded View Figures

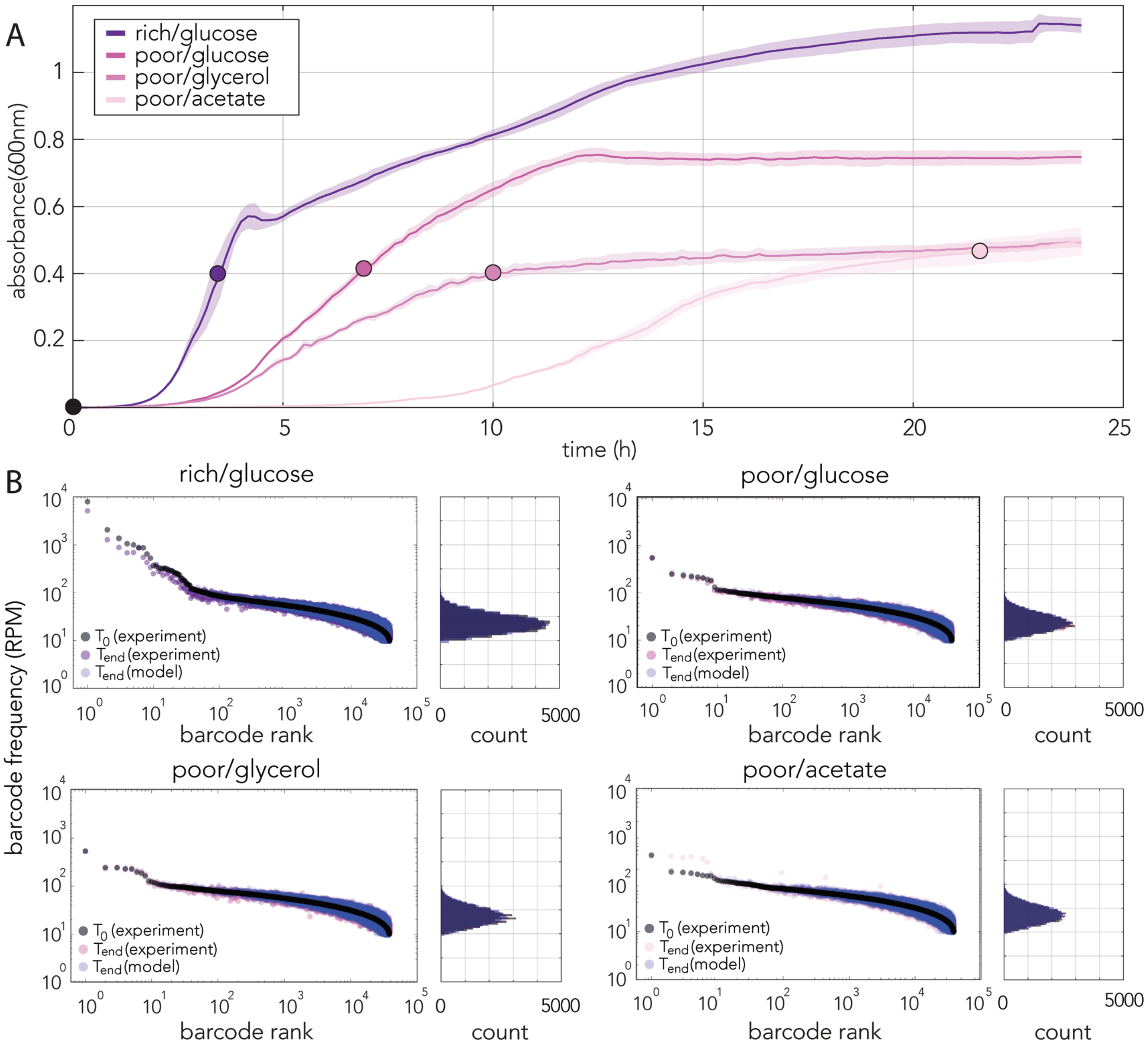

Figure EV1. Variation in barcode frequency after in vitro growth follows the Monod model with intrinsic noise.

(**A**) Growth of barcoded strain collection and isolation points for barcode extraction. Error shades represent standard deviation. (**B**) Variation in barcode frequency before and after growth. The frequency of barcodes in the inoculum is shown and compared to experiment and model simulation results without reranking to show growth noise (see Methods for in vitro model). Intrinsic noise does not change the relative frequency of barcodes; reranking results in the same rank-frequency distribution as the inoculum. Data information: Data shown from a single experiment. Technical replicates $n = 4$ for (**A**), and $n = 3$ for (**B**).

 © The Author(s)

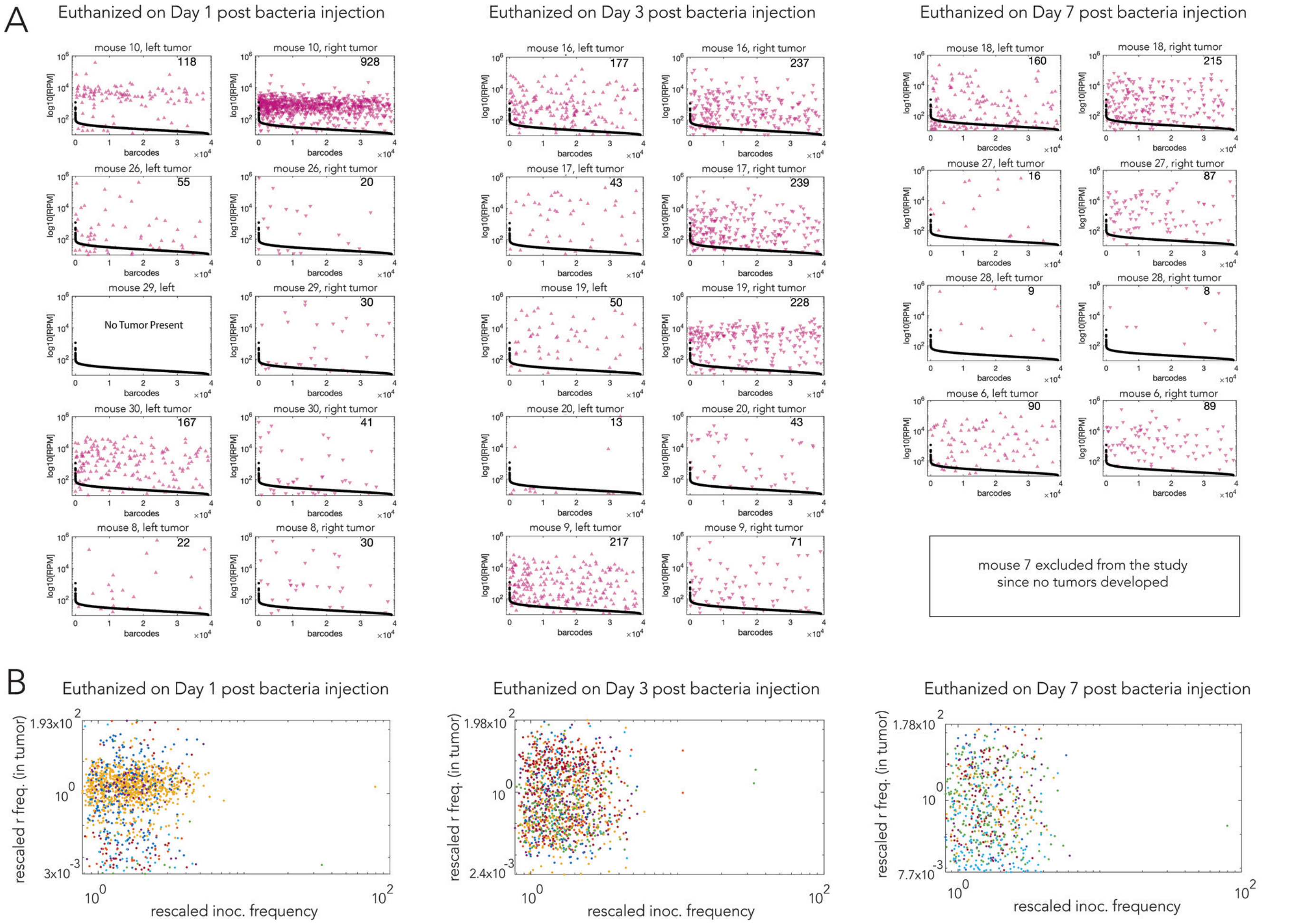

Figure EV2. **(A)** Frequency of detected unique barcodes from left and right tumors of individual mice from the i.v. injected condition grouped by day of euthanasia. Detected barcodes were ranked in decreasing frequency in the inoculum. The number in the upper right corner of each panel indicates the total number of unique barcodes in the tumor. **(B)** No correlation is found between the inoculum frequency and the frequency post i.v. injection for all days as shown in the last panel of each column (points in different colors mark different tumors). Spearman correlation coefficients were all around 0 [−0.02–0.02]. Data information: Data from a single experiment. Number of total mice = 14, technical replicates $n = 2$.

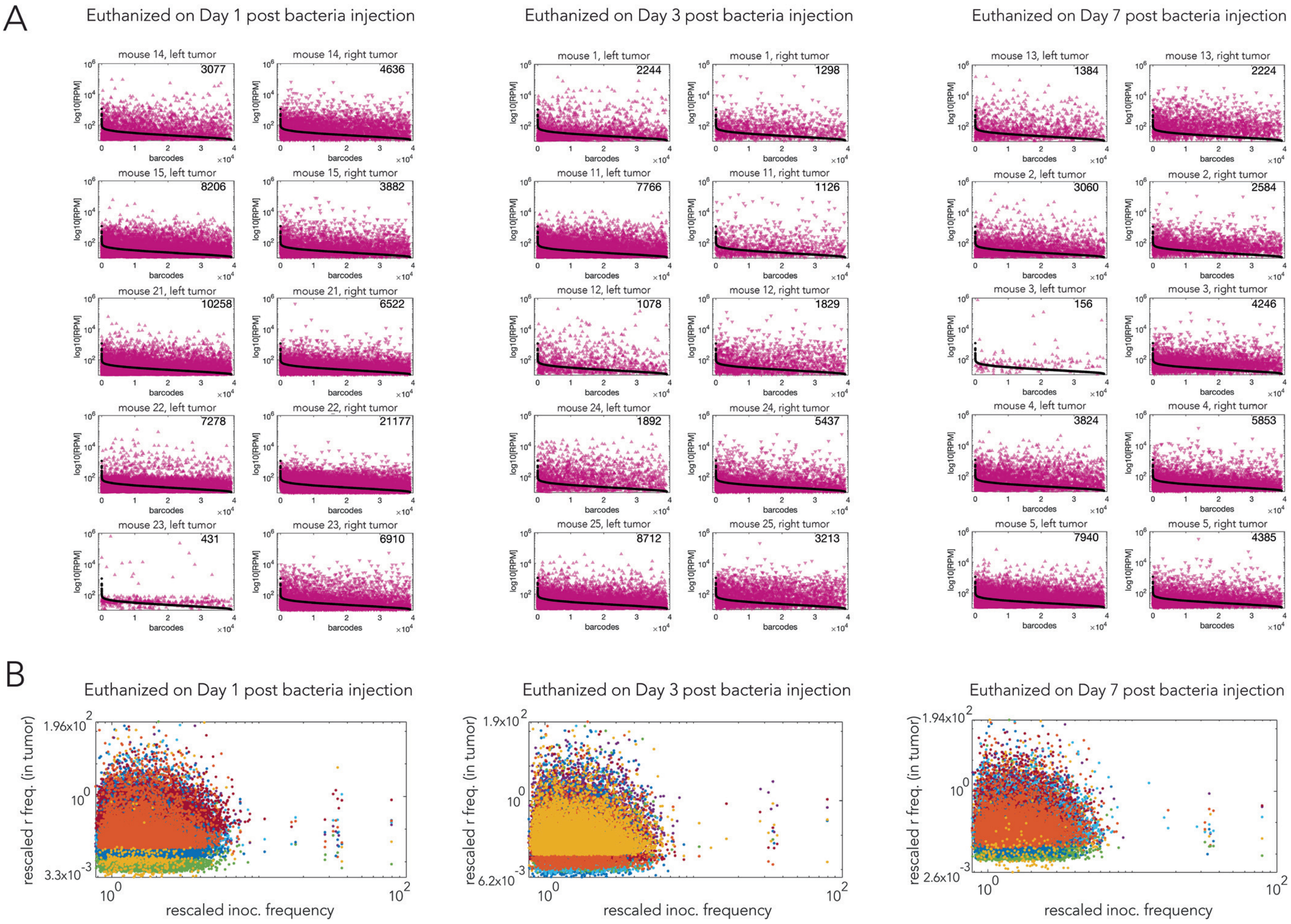

Figure EV3. **(A)** Frequency of detected unique barcodes from left and right tumors of individual mice from the i.t. injected condition, grouped by day of euthanasia. Detected barcodes were ranked by decreasing frequency in the inoculum. The number in the upper right corner of each panel indicates the total number of unique barcodes in the tumor. Number of total mice = 14, technical replicates $n = 2$. **(B)** No correlation is found between the inoculum frequency and the frequency post i.t. injection for all days, as shown in the last panel of each column (points in different colors mark different tumors). Spearman correlation coefficients were all around 0 [−0.02–0.02]. Data information: Data from a single experiment. Number of total mice = 15, technical replicates $n = 2$.

 © The Author(s)

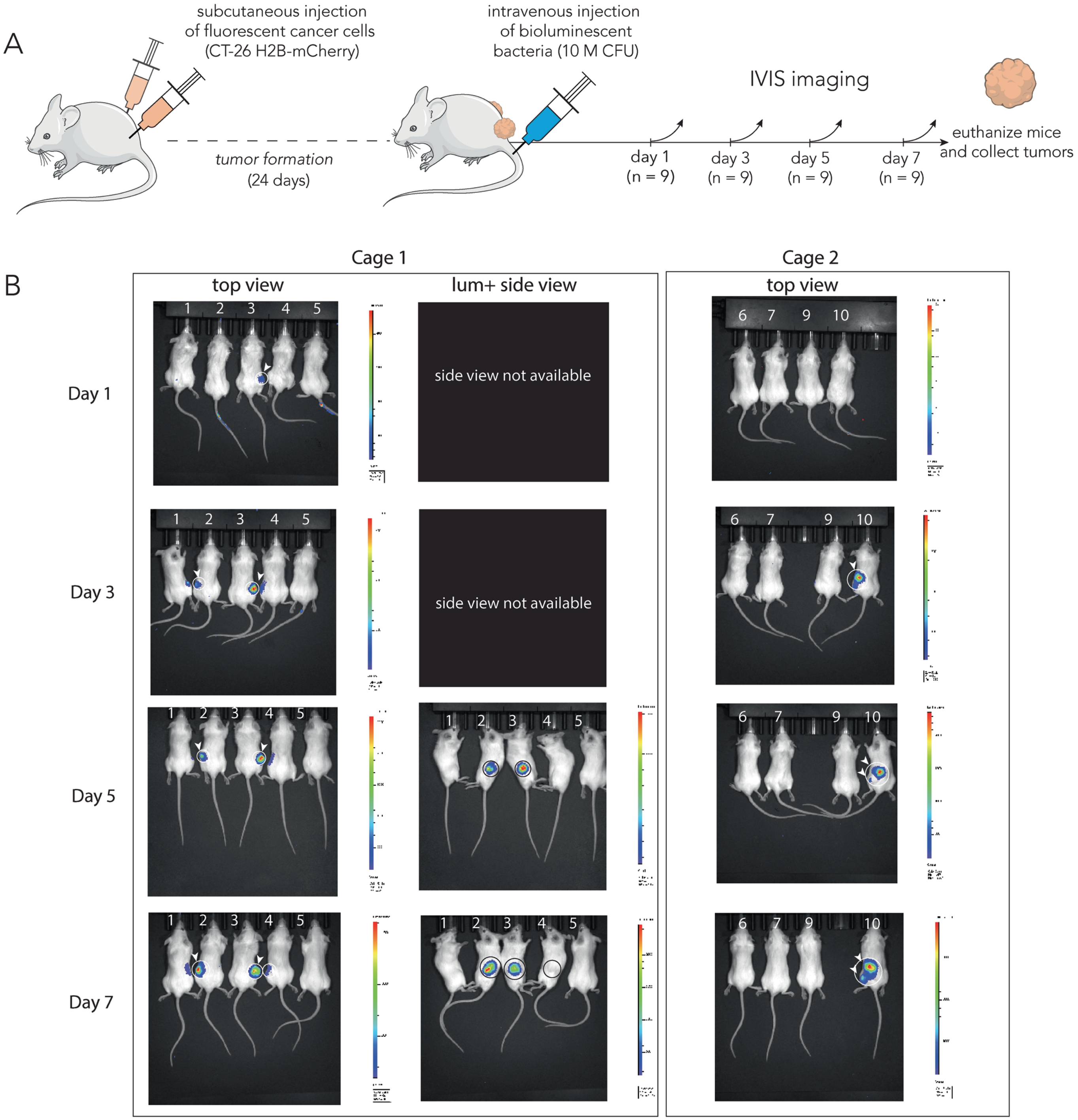

Figure EV4. Bacteria localize exclusively to tumors.

(**A**) Outline of experimental approach. Subcutaneous tumors were formed on the right and left flanks by injection of CT-26 cells and allowed to grow for 24 days. Tumors developed in four of the nine mice. All mice were injected intravenously with ten million CFUs of bioluminescent *E. coli* Nissle 1917. (**B**) Bioluminescent images of the mice were captured on days 1, 3, 5, and 7 post bacteria injection. Circles mark the location of developed tumors, and arrows mark a clear bioluminescent signal. A bioluminescent signal was observed exclusively in the tumor area (3 of 4 mice). In one mouse with a tumor, mice 4 of cage 1, bacterial colonization failed (1 of 4). We did not observe any bacteria in other organs in these mice (besides the initial wound from tail injection on day 1), and we did not observe bacteria in mice that did not have a developed tumor (5 of 5). Data information: Data from a single experiment. Number of total mice = 9.

© The Author(s)
